# Isolation and Molecular Characterization of Emerging Getah Virus Genotype III Variant Strains from Swine in Southeastern China (2024–2025)

**DOI:** 10.3390/ijms27136016

**Published:** 2026-07-04

**Authors:** Xiufang Yuan, Bin Yu, Xingyuan Ma, Lihua Xu, Fei Su, Hongchao Sun, Kang Shao, Tao Xiong, Junxing Li, Shiyi Ye

**Affiliations:** 1 Institute of Animal Husbandry and Veterinary Science, Zhejiang Academy of Agricultural Sciences, Hangzhou 310021, China; yuanxf@zaas.ac.cn (X.Y.); yub@zaas.ac.cn (B.Y.); 2024710979@yangtzeu.edu.cn (X.M.); xulihua@zaas.ac.cn (L.X.); sufei@zaas.ac.cn (F.S.); sunhongchao1988@126.com (H.S.); lijunx@zaas.ac.cn (J.L.); 2 College of Life Science, Yangtze University, Jingzhou 434025, China; xiongtao@hotmail.com; 3 College of Chemical Engineering, Zhejiang University of Technology, Hangzhou 310014, China; sk1033@zjut.edu.cn

**Keywords:** Getah virus, variant strain, genotype III, phylogenetic analysis, epidemiology, pathogenicity

## Abstract

As a member of the Alphavirus genus, Getah virus (GETV) has become an emerging pathogen posing severe threats to animal health and public health security. In recent years, GETV has caused increasing outbreaks in swine herds, leading to substantial economic losses in the pig industry in China. Here, we report continuous GETV outbreaks on pig farms across southeastern China between September 2024 and December 2025. A total of 185 clinical samples were examined, among which 27 were positive (14.6%). Subsequently, five GETV strains were successfully isolated and subjected to whole-genome sequencing. Phylogenetic analysis revealed that all the 5 isolated strains belonged to genotype III(GIII), with two strains (GETV-AHX2024 and GETV-JS2024) forming a distinct GIII variant clade. Notably, multiple specific amino acid mutations were identified in the nsp3, C, E2, 6K, and E1 proteins and 3′UTR of these GIII variant strains. Pathogenicity in mice revealed that mice infected with the GIII variant strain GETV-JS2024 exhibited a longer viral shedding period compared with other strains. These findings highlight the emergence of GETV GIII variant strains in southeastern China and suggest their altered pathogenic performance in mice. These results supplied critical data for molecular surveillance and subsequent pathogenic analysis of GETV in China.

## 1. Introduction

Getah virus (GETV) is a mosquito-borne zoonotic virus belonging to the Alphavirus genus of the Togaviridae family [1,2]. GETV was first isolated from Culex mosquitoes in Malaysia in 1955 [1,3]. At present, GETV has been reported in numerous countries across Eurasia and the Pan-Pacific, including China, Korea, Japan, Russia, Thailand, Mongolia, India, Australia, Philippines, Sri Lanka and Vietnam [4,5,6,7].

GETV has a broad host range, infecting various domestic and wild animals, including pigs, horses, cattle, foxes, red pandas, and wild boars [8,9,10,11,12,13,14]. It is worth noting that GETV antibodies have been detected in human serum [15,16]; however, no confirmed cases of clinical GETV-associated disease in humans have been reported to date. Such seropositivity indicates human exposure to GETV, while its pathogenic potential to humans and corresponding public health risks remain unclear and require further investigation.

The genome of GETV is single-stranded positive-sense RNA, approximately 11–12 kb in length. The GETV genome consists of a 5′ untranslated region (UTR), two open reading frames (ORF1 and ORF2), and a 3′ UTR. ORF1 and ORF2 encode four nonstructural proteins (nsp1–nsp4) and five structural proteins (C, E3, E2, 6K, and E1), respectively [17,18]. Based on genomic sequence variations, GETV can be classified into four groups (GI–GIV). GIII is currently the predominant genotype and poses the greatest threat to animal health, particularly in East Asia.

In recent years, reports of GETV infection in swine have continuously increased, causing significant economic losses to the pig industry [19]. The porcine GETV infection showed obvious age-related characteristics. Newborn piglets are the most susceptible [20]. Porcine infection showed diverse clinical manifestations, including fever, diarrhea, anorexia, skin congestion, and reproductive failure, leading to a high mortality rate in piglets [21,22]. Furthermore, studies have identified GETV contamination in commercial live vaccines for pigs [23,24], suggesting that vaccination could be a potential route for GETV transmission.

In this study, five GETV strains were isolated from clinical samples collected from pig farms in Zhejiang, Anhui and Jiangsu provinces during 2024–2025. Whole-genome sequencing and phylogenetic analysis identified two strains as novel GIII variant strains carrying signature mutations in structural and non-structural genes. Mouse infection experiments demonstrated that the variant strain GETV-JS2024 exhibited prolonged viral shedding. Our results provide critical insights into the molecular evolution and pathogenicity of emerging GETV variant strains in southeastern China.

## 2. Results

### 2.1. The Prevalence of GETV in Southeastern China

**Figure 1:**
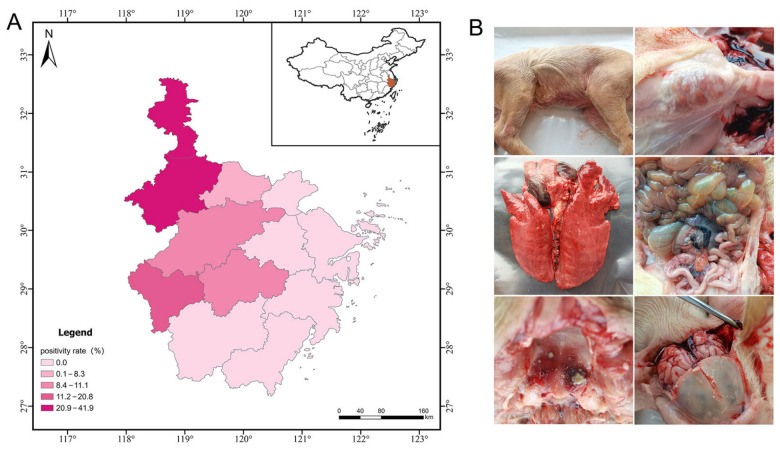
Epidemic information of GETV. (**A**), The prevalence of GETV in Zhejiang province and its surrounding areas. (**B**), Gross tissue lesions of infected piglets in this outbreak.

From 2024 to 2025, many pig farms in Zhejiang and its surrounding areas suffered diarrhea and death in piglets. A total of 185 clinical samples suspected of GETV were tested, and 27 were GETV-positive (14.6%), including 10 tissues from aborted fetuses and 17 tissues from piglets. The statistical results showed that GETV epidemics were observed in the junction of Zhejiang, Anhui and Jiangsu provinces (Figure 1A and Table S1).

Necropsy of the dead pigs was performed in our lab. The lungs, lymph nodes, kidneys and other organs were collected for viral pathogen detection and pathological analysis. The results of necropsy showed that the GETV-infected piglets exhibited lesions in multiple organs, including cyanosis of the skin, swollen lymph nodes, pulmonary hemorrhage, and thinned intestinal wall (Figure 1B). The qPCR results showed that the samples in this farm were GETV positive and negative for other porcine origin viruses (porcine reproductive and respiratory syndrome virus [PRRSV], classical swine fever virus [CSFV], pseudorabies virus [PRV], porcine epidemic diarrhea virus [PEDV], porcine deltacoronavirus [PDCoV], porcine transmissible gastroenteritis virus [TGEV], and porcine circovirus type 2 [PCV2]). These results indicated that GETV is the causative pathogen for the lesions. Furthermore, the qPCR results of different organs showed that GETV could be detected in the lungs, liver, spleen, kidney, brain, lymph nodes and intestinal fluid.

### 2.2. GETV Isolation and Identification

**Figure 2:**
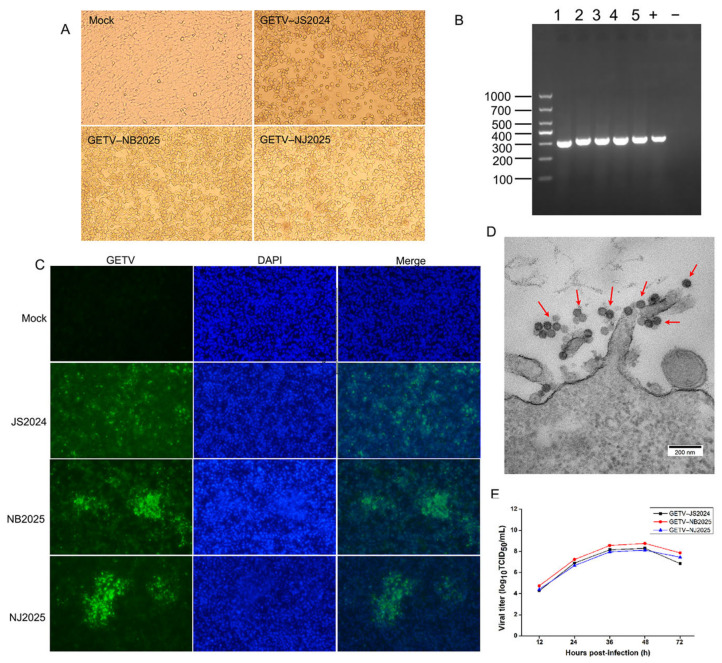
Isolation and identification of GETV. (**A**) CPEs of GETV strains on BHK-21 cells. (**B**) PCR identification of GETV strains. (**C**) Immunofluorescence was used to illustrate the presence of GETV strains in BHK-21 cells. The fluorescence images were acquired at a 20-time magnification. (**D**) Viral particles of GETV were observed by transmission electron microscopy. The viral particles are marked with red arrows. (**E**) Proliferation of GETV strains on BHK-21 cells.

Five GETV strains were successfully isolated from piglet lung or aborted fetus tissues. All the GETV strains exhibited significant cytopathic effect (CPE) characterized by cell rounding, shrinkage and extensive detachment on BHK-21 cells (Figure 2A and Figure S1), while no CPE was observed in control cells. RT-PCR analysis showed a 316 bp amplification product, confirming GETV infection (Figure 2B). As shown in Figure 2C, immunofluorescence microscopy revealed robust signals, indicating effective infection of GETV-JS2024 and GETV-NB2025, along with the remaining three strains (Figure 2C and Figure S2). GETV particles about 70 nm in diameter (Figure 2D) were observed by ultrathin cell sections.

To evaluate the replication dynamics of the GETV strains, viral replication kinetics were analyzed in BHK-21 cells. The viral proliferation curves of three representative GETV strains (GETV-JS2024, GETV-NB2025 and GETV-NJ2025) were determined on BHK-21 cells at a multiplicity of infection of 0.1. These three strains reached peak viral titers at approximately 48 h post-infection. Notably, the GETV-NB2025 strain exhibited the highest viral titer, peaking at 10^8.75^ TCID_50_/mL (Figure 2E).

### 2.3. Sequencing and Phylogenetic Analysis

The 5 GETV isolations were sequenced, and their whole-genome sequences were obtained (GenBank accession numbers: PZ445640-PZ445644). As shown in Table S2, the genomes of the strains were 11,689–11,695 bp in length. The genomes of GETV strains were arranged as 5′ UTR-nsp1-nsp2-nsp3-nsp4-C-E3-E2-6K-E1-3′UTR, which is consistent with other strains of GETV.

**Figure 3:**
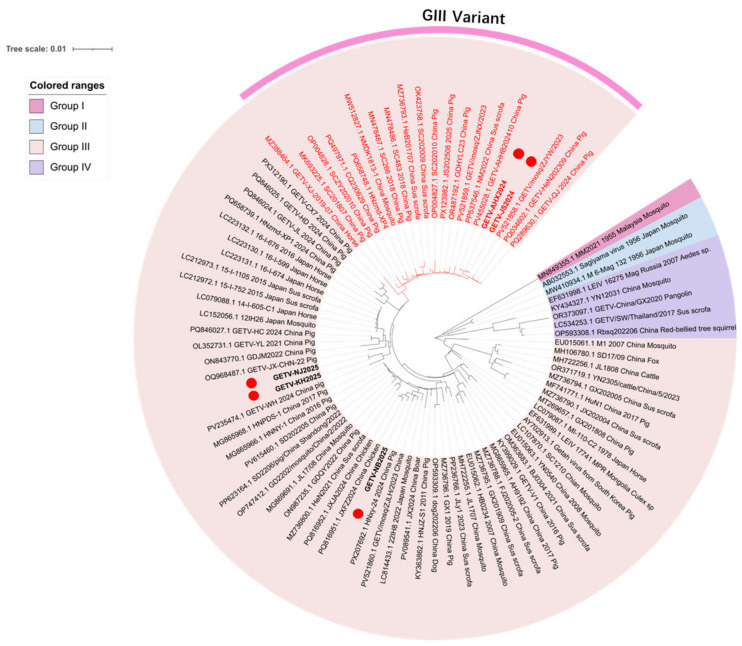
Phylogenetic analysis of GETV strains based on complete genomes. The GETV strains obtained and sequenced in this study are represented by red dots. The GIII variant strains are indicated in red font.

Phylogenetic analysis was constructed based on the whole genomes of GETV. All the GETV strains isolated in this study belong to Group III (Figure 3). Phylogenetic analysis confirms that the GETV-NB2025 strain has the closest genetic relationship with the JXF2024 strain isolated from chickens and the HNxy-24 strain derived from pigs. GETV-NJ2025 and GETV-KH2025 have the closest genetic relationship with the GETV-WH strain, which was derived from pigs in 2024. GETV-AHX2024 and GETV-JS2024 isolates have the closest genetic relationship with the GETV/mosq/JZYW/2023 strain that originated from mosquitoes and the AHHB202410 strain from Pigs.

The phylogenetic tree showed that the strains GETV-AHX2024 and GETV-JS2024 were closest to each other, as well as the other two strains, GETV-KH2025 and GETV-NJ2025. Notably, GETV-AHX2024 and GETV-JS2024 formed a distinct subclade together with recent Chinese strains (GDHYLC23, GETV-QJ), designated the GIII variant clade [25,26].

**Figure 4:**
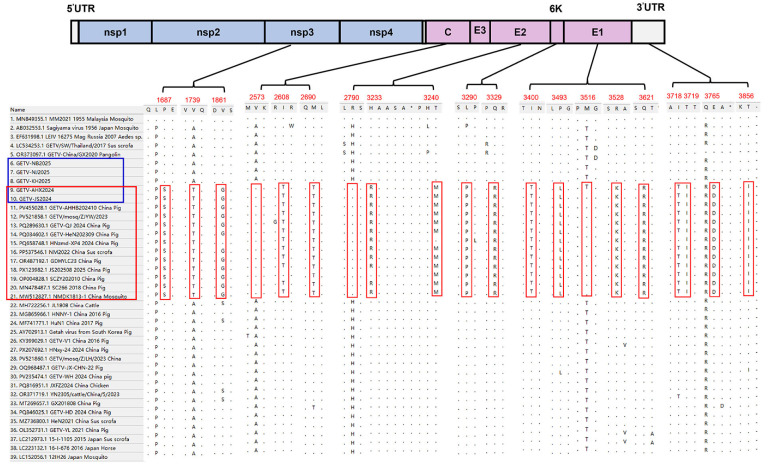
Amino acid sequence alignment based on the GETV complete genome. The GIII variant strains are in the red frame; The strains isolated in this study are represented by a blue frame; Specific amino acid mutations of the GIII variant that differ from other GETV GIII strains are highlighted in red boxes.

The amino acid sequence alignment of GETV revealed that the GETV-AHX2024 and GETV-JS2024 strains, together with GIII variant strains, have some characteristic mutations in nsp3, C, E2, 6K, E1 and 3′ UTRs (Figure 4). The mutations in nsp3 were consistent with those reported by Sun et al. [26]. Cap protein (C) assembles into the viral capsid, playing an essential role in virus infection [27]. Specifically, the C protein has 3 amino acid mutations at position 2573 (Ala^2573^Val), 2608 (Ile^2608^Thr) and 2690 (Met^2690^Thr). The mutations in E2 were at positions 3233 and 3240, which change Histidine to arginine and threonine to methionine, respectively. The mutations in 6K, E1 and 3′UTRs were shown in Figure 4. Based on these findings, we determined that GIII strains have evolved into a new variant branch.

### 2.4. Pathogenicity of GETV Strains in Mice

**Figure 5:**
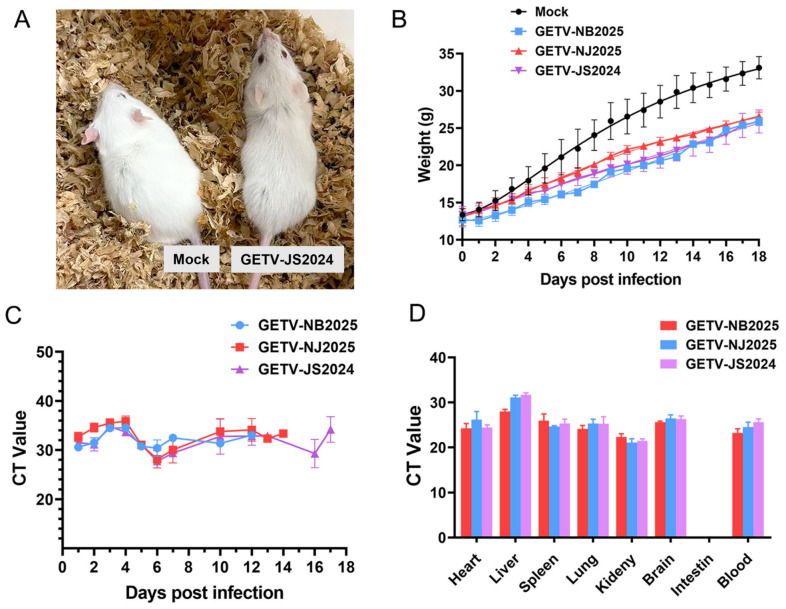
Pathogenicity of GETV strains in mice. (**A**) Clinical signs of infected and mock mice. (**B**) Weight change in mice in different groups. (**C**) GETV detection of feces in each infected group. (**D**) GETV detection in different organs in each infected group.

Previous studies demonstrated that mice could serve as optimal models for investigating the virulence of GETV [26,28,29]. GETV-JS2024, GETV-NB2025 and GETV-KH2025 were selected as three distinct strains to infect mice. Results from the infected mice showed that the three challenge groups exhibited growth retardation and hypoactivity (Figure 5A,B). The feces of mice in each group were collected every day and subjected to qPCR to monitor virus elimination. Results showed that GETV could be detected in the feces of all infected mice starting from 1 day after infection (Figure 5C). The mice in the GETV-JS2024 group continued to excrete the virus for 17 days, longer than GETV-NJ2025 (14 days) and GETV-NB2025 (12 days) (Figure 5C). After 7 days post-infection, 3 mice in each group were sacrificed. Organs (hearts, lungs, livers, spleens, kidneys, intestines, brains) and blood were collected for GETV detection by qPCR. As shown in Figure 5D, GETV was positive in all infected organs except the small intestine, suggesting that GETV has a wide range of tissue distribution. Body weight reduction and duration of viral shedding via feces indicated GETV pathogenicity in mice, as reported in previous studies [26,28]. These results indicated that the GETV-JS2024 strain exhibits stronger pathogenicity in mice.

## 3. Discussion

As a mosquito-borne virus, GETV is continuously threatening global livestock and public health security. In recent years, reports of GETV infection in pig farms have been continuously increasing in China, causing a new threat to the pig industry. The economic losses caused by GETV to the pig industry in recent years remind us that we should attach great importance to the monitoring of GETV.

Here, we report the isolation and characterization of five GETV GIII strains from swine in southeastern China during 2024–2025, including two novel GIII variant strains with signature mutations and enhanced shedding in mice. Since 2018, swine GETV outbreaks have re-emerged in multiple Chinese provinces, refs. [30,31,32,33] coinciding with the spread of GIII strains. Our strains are closely related to recent Chinese strains from pigs, chickens, and mosquitoes, indicating active local circulation and cross-species transmission potential.

Studies have shown that the genetic evolution rate of GETV over the past 150 years has been significantly higher than that of other members of the genus alphavirus [34]. This highly variable characteristic increases the potential risk of it adapting to new hosts and environments as well as breaking through immune barriers. It is worth noting that recent studies have found that, unlike previous Group III strains, strains isolated in the past two years such as GDHYLC23 and HN1 have formed a new branch (i.e., the GETV GIII variant strain), which shows stronger virulence to mice and pigs [21,25,26]. The GETV-AHX2024 and GETV-JS2024 strains isolated in this study also cluster within this variant clade. Combined with our mouse infection data and previous research, we hypothesize that the pathogenicity of currently circulating GETV strains may have increased.

The emergence of GIII variant strains carrying mutations in nsp3, C, and E2 is particularly noteworthy. These characteristic amino acid mutations provide compelling molecular evidence for the ongoing adaptive evolution of GETV. The nsP3 protein is critical for viral replication complex formation, which is closely associated with viral replication [35]. Capsid (C) and E2 are major structural proteins involved in virion assembly and host cell entry [27,36]. Mutations in these regions may enhance viral fitness, adaptation to swine hosts, or evasion of host immune responses. Although these mutations in nsp3, C, E2, 6K, E1 and the 3′UTR could serve as characteristic molecular signatures that distinguish GETV GIII variant strains from other genotypes, their functional contributions to viral replication, host adaptation and virulence remain unvalidated. The correlation between these mutations and viral phenotypic differences is therefore tentative and requires further functional verification.

Recent studies have shown that GIII variant strains exhibit higher virulence in pigs and mice than classical GIII strains [26], consistent with our finding that the GETV-JS2024 variant strain induced prolonged shedding in mice. These results preliminarily suggested that the GETV-JS2024 variant strain may exhibit increased pathogenicity in the mouse model. However, its pathogenicity in swine requires further investigation. Prolonged viral shedding increases the risk of persistent horizontal transmission within farms via contaminated surroundings, which may sustain and expand on-farm outbreaks. This phenotypic difference may correlate with enhanced viral fitness or altered host interaction mechanisms, warranting further investigation into its underlying molecular determinants. Given the high evolutionary rate of GETV and its potential capacity for increased virulence, sustained monitoring is essential to track variant spread and evolution. The detection of GETV in aborted fetuses and vaccine contamination reports highlight the need for improved biosecurity, vector control, and vaccine quality surveillance.

## 4. Materials and Methods

### 4.1. Sample Collection and Detection

During September 2024 to December 2025, clinical samples suspected GETV infection were collected from aborted fetuses and piglets with suspected GETV infection on pig farms in southeastern China. The criteria for inclusion of samples were determined based on the epidemiological characteristics of GETV infection. Samples from suckling piglets in farrowing houses presenting fever, diarrhea or neurological symptoms, as well as aborted fetuses and stillbirths with corresponding epidemiological features, were included in sample statistics. A total of 185 samples were collected from 52 pig farms in Zhejiang, Jiangsu and Anhui provinces. The collected tissues included lungs, lymph nodes, livers, spleens, hearts, kidneys, brains, intestines, and umbilical cords.

The collected tissues were dissected into small pieces and placed in 2 mL RNA-free tubes. Then, an appropriate amount of PBS was added, followed by full homogenization using a tissue homogenizer. Viral RNA was extracted according to the manufacturer’s instructions. All samples were screened for GETV and other common swine pathogens (PRRSV, CSFV, PRV, PEDV, PDCoV, TGEV, PCV2) by quantitative real-time PCR (qPCR). Positive samples were stored at −80 °C for further use.

### 4.2. Isolation and Identification of GETV

The homogenized tissues of the positive samples were centrifuged at 12,000 rpm for 10 min. The supernatants were collected and filtered through a 0.22 μm filter. After being diluted in Dulbecco’s Modified Eagle Medium (DMEM, Gibco), the samples were inoculated into BHK-21 cells for 1 h at 37 °C in a CO_2_ incubator. Then, the inoculum was discarded, and the cells were maintained in DMEM containing 2% fetal bovine serum and penicillin-streptomycin. Cells were harvested in the presence of obvious cytopathic effect (CPE), and successive passages were performed. Three rounds of plaque purification assay were implemented to purify the virus when obvious CPE was monitored. The isolated GETV strains were identified by RT-PCR and sequencing.

### 4.3. Biological Characterization of GETV

The GETV strains were inoculated onto BHK-21 in 24-well plates. After incubation for 1 h, the cells were washed twice with PBS and then maintained in DMEM containing 2% FBS. The cells with obvious CPE were washed and fixed with 4% paraformaldehyde for 20 min at room temperature. After washing with PBS 3 times, the fixed cells were used for immunofluorescence. Briefly, a 1:100 dilution of GETV antibody (derived from infected recovered pigs) was added and incubated at 37 °C for 1 h. Finally, FITC-conjugated goat anti-pig IgG was incubated at 37 °C for 45min. The nucleus of the cells was then stained with DAPI at room temperature for 10min, and finally observed under an inverted fluorescence microscope (Olympus, Tokyo, Japan).

The viral titers of GETV were determined by the 50% tissue culture infectious dose (TCID_50_) assay. To determine the growth kinetics curve of the GETV strains, viruses were inoculated onto BHK-21 in 6-well plates at an MOI of 0.1. The cell culture was collected at 12, 24, 36, 48, and 72 h post-infection (hpi). The TCID_50_ of GETV was calculated using the Reed-Muench method [37].

The virus was cultured on BHK-21 cells to propagate in a T75 culture flask. The cells were washed and digested with trypsin when the cells exhibited 20–50% CPE. The cells were collected by centrifugation at 1000 rpm for 5 min. After being rinsed twice with PBS, the cells were fixed with 2.5% glutaraldehyde solution overnight at 4 °C. Subsequently, the cells were rinsed 3 times with PBS and fixed with precooled 1% osmium acid. The cells were then gradually dehydrated in 30%, 50%, 70%, 90%, and 100% ethanol. Afterward, the cells were penetrated and embedded with epoxy resin. The samples were then ultrathin sectioned with an ultramicrotome (Leica, Wetzlar, Germany). After being stained with 2% uranyl acetate and 0.2% lead citrate, the ultrathin sections were then dried naturally and observed using a 100-kV transmission electron microscope (Hitachi, Tokyo, Japan).

### 4.4. Genome Sequencing and Phylogenetic Analysis

To construct a phylogenetic tree of GETV, a total of 81 whole-genome sequences were obtained from NCBI (Supplementary Tables S3). The strains utilized in this analysis were derived from multiple countries, such as China, Russia, Japan, Korea, Thailand, Malaysia and Mongolia, and represented a range of host species. Sequence alignments and phylogenetic analysis were performed using MEGA 11. The phylogenetic tree was generated using the maximum likelihood (ML) method and modified by iTOL. Bootstrap values are represented as a percentage based on 1000 replicates.

### 4.5. Pathogenicity in Mice

All animal experiments were performed and approved by the Experimental Animal Welfare Ethics Committee of Zhejiang academy of agricultural sciences. Twenty-four 3-week-old female balb/c mice were randomly divided into 4 groups (mock, GETV-NB2025, GETV-NJ2025 and GETV-JS2024), with 6 mice in each group. Mice in 3 challenge groups received a subcutaneous injection of 10^7^ TCID_50_ of the GETV strains. Mice in the mock group were injected with DMEM. All mice were maintained at a temperature of 25 ± 5 °C, with a 12 h light-dark cycle. The mice were observed and weighed every day. The mice were placed separately in a breathable jar to collect the feces. Three mice in each group were sacrificed to collect the organs (heart, liver, lung, spleen, kidney, small intestine and brain) and blood at 7 days post-infection. GETV was detected in each organ by qPCR. Additionally, the fourth author was blinded to group assignment during animal feeding and sample collection.

Statistical analyses were performed using GraphPad Prism 8 (Version: 8.0.2). In the mouse infection experiment, there were six biological replicates in each group within the first 7 days of infection, and then three replicates in each group thereafter. Data are presented as means ± SDs.

## 5. Conclusions

In summary, we successfully isolated and characterized 5 GETV GIII strains from piglets in southeastern China between 2024 and 2025, including two novel GIII variant strains with characteristic mutations in nsp3, C, E2, 6K, E1, and 3′UTR. The GIII variant GETV-JS2024 exhibited a prolonged viral shedding period in mice, suggesting possible enhanced transmission potential. These results provide critical data on the molecular epidemiology and pathogenicity of emerging GETV GIII variant strains, supporting ongoing surveillance and control efforts.

## Data Availability

The sequences of GETV strains isolated in this study have been publicly deposited in the National Center for Biotechnology Information (NCBI) (GenBank accession numbers: PZ445640-PZ445644). The raw datasets supporting the conclusions of this study are available on request from the authors.

## Supplementary Materials

The following supporting information can be downloaded at: https://www.mdpi.com/article/10.3390/ijms27136016/s1.
