# Supplementary material for: Isolation and Molecular Characterization of Emerging Getah Virus Genotype III Variant Strains from Swine in Southeastern China (2024–2025)
Source: Int J Mol Sci. 2026 Jul 4;27(13):6016. doi: 10.3390/ijms27136016 (PMC13361111; doi:10.3390/ijms27136016)
Supplement: Supplementary file 1 [file ijms-27-06016-s001.zip › ijms-4371738-supplementary.pdf]

Supplementary Materials

Isolation and Molecular Characterization of Emerging Getah Virus Genotype III Variant Strains from Swine in Southeastern China (2024–2025)

Xiufang Yuan [1,†], Bin Yu [1,†], Xingyuan Ma [1,2], Lihua Xu [1], Fei Su [1], Hongchao Sun [1], Kang Shao [3], Tao Xiong [2], Junxing Li [1] and Shiyi Ye [1,*]

[1] Institute of Animal Husbandry and Veterinary Science, Zhejiang Academy of Agricultural Sciences, Hangzhou 310021, China; yuanxf@zaas.ac.cn (X.Y.); yub@zaas.ac.cn (B.Y.); 2024710979@yangtzeu.edu.cn (X.M.); xulihua@zaas.ac.cn (L.X.); sufei@zaas.ac.cn (F.S.); sunhongchao1988@126.com (H.S.); lijunx@zaas.ac.cn (J.L.)
[2] College of Life Science, Yangtze University, Jingzhou 434025, China; xiongtao@hotmail.com
[3] College of Chemical Engineering, Zhejiang University of Technology, Hangzhou 310014, China; sk1033@zjut.edu.cn
* Correspondence: yesy@zaas.ac.cn
† These authors contributed equally to this work.

Table S1 Epidemiological Information of Getah virus infections in different cities in Southeastern Chian between 2024 and 2025

Location	Date and Farm Distribution	Samples	Positive Samples	Animal age	Clinical signs	Total Samples	Total Positive Samples	Positive Rate(%)
Hangzhou	24.12 farm16	2	0	aborted fetus and sow blood	reproductive disorders	10	1	10
	24.12 farm18	1	1	7 d	Fever, diarrhea and death			
	24.12 farm19	1	0	piglet feces	diarrhea			
	25.3 farm29	3	0	piglet	diarrhea, emaciation			
	25.8 farm41	1	0	aborted fetus	reproductive disorders			
	25.9 farm44	2	0	piglet	diarrhea			
Huzhou	24.10 farm12	2	0	piglet	Not available	12	1	8.33
	25.1 farm22	1	0	aborted fetus and sow blood	reproductive disorders			
	25.3 farm28	3	0	7-10 d	fever and diarrhea			
	25.4 farm36	2	0	piglet	Not available			
	25.7 farm38	1	1	7 d	fever and diarrhea			
	25.8 farm22	2	0	aborted fetus	reproductive disorders			
	25.11 farm28	1	0	aborted fetus	reproductive disorders			
Jiaxing	25.3 farm32	5	0	10-15 d	diarrhea	15	0	0
	25.10 farm47	10	0	Piglet feces	diarrhea			

Shaoxing	24.9 farm5	1	0	aborted fetus	reproductive disorders	21	0	0
	24.10 farm8	1	0	aborted fetus	reproductive disorders			
	24.10 farm9	1	0	piglet	Not available			
	25.1 farm21	2	0	aborted fetus and sow blood	reproductive disorders			
	25.4 farm21	2	0	piglet	Not available			
	25.5 farm36	4	0	aborted fetus	reproductive disorders			
	25.6 farm36	1	0	aborted fetus	reproductive disorders			
	25.7 farm37	3	0	aborted fetus	reproductive disorders			
	25.7 farm36	1	0	piglet	Not available			
	25.7 farm40	1	0	aborted fetus	reproductive disorders			
	25.9 farm40	2	0	aborted fetus	reproductive disorders			
	25.12 farm52	2	0	piglet	Not available			
Ningbo	24.10 farm10	2	0	aborted fetus	reproductive disorders	10	0	0
	24.10 farm13	1	0	piglet	fever and diarrhea			
	24.11 farm13	2	0	piglet	vomiting and diarrhea			

	25.4 farm13	3	0	piglet	Not available			
	25.9 farm46	2	0	piglet feces	diarrhea			
Zhoushan	24.11 farm14	5	0	aborted fetus	reproductive disorders	5	0	0
Qvzhou	24.9 farm3	2	2	7 d	fever and diarrhea	24	5	20.83
	24.9 farm4	1	0	aborted fetus	reproductive disorders			
	24.9 farm6	2	0	piglet	diarrhea			
	24.10 farm11	1	1	piglet	diarrhea			
	24.11 farm15	1	1	piglet	fever and death			
	24.12 farm17	2	0	aborted fetus and sow blood	reproductive disorders			
	24.12 farm20	1	0	aborted fetus	reproductive disorders			
	25.1 farm24	2	0	piglet	Not available			
	25.2 farm27	1	0	3 d	diarrhea			
	25.3 farm31	3	0	piglet feces	diarrhea			
	25.4 farm34	2	0	aborted fetus	reproductive disorders			
	25.4 farm3	1	0	piglet feces	diarrhea			
	25.4 farm35	3	1	piglet and feces	Diarrhea			
	25.5 farm31	1	0	feces	Diarrhea			
	25.7 farm31	1	0	feces	diarrhea			
Jinhua	24.9 farm7	2	0	piglet	Not available	18	2	11.11
	25.2 farm26	3	0	piglet	diarrhea			

	25.3 farm33	5	0	sow blood	reproductive disorders			
	25.4 farm33	4	0	aborted fetus and sow blood	reproductive disorders			
	25.10 farm48	2	0	aborted fetus	reproductive disorders			
	25.10 farm49	2	2	7-10 d	fever, vomiting and diarrhea			
Taizhou	25.11 farm50	3	0	piglet	diarrhea, emaciation	8	0	0
	25.12 farm51	5	0	10-13 d	fever and diarrhea			
Lishui	25.2 farm25	3	0	1 aborted fetus and 2 sow blood	reproductive disorders	9	0	0
	25.7 farm39	2	0	piglet	Not available			
	25.8 farm42	4	0	piglet feces	diarrhea			
Wenzhou	25.3 farm28	3	0	piglet feces	Diarrhea	8	0	0
	25.9 farm43	5	0	aborted fetus	reproductive disorders			
Nanjing	24.9 farm1	5	2	10 d	fever, vomit and diarrhea	14	5	35.71
	24.9 farm2	6	3	7 d	Fever, diarrhea and death			
	25.3 farm30	3	0	piglet	vomit and diarrhea			
Xuancheng	25.1 farm23	13	3	piglet feces	vomit and diarrhea	31	13	41.94
	25.7 farm38	2	2	dead fetus	reproductive disorders			

	25.7 farm23	8	8	12-13 d	fever, diarrhea and death			
	25.9 farm45	3	0	piglet blood	fever, diarrhea			
	25.10 farm23	5	0	aborted fetus and sow blood	reproductive disorders			
Total						185	27	14.6

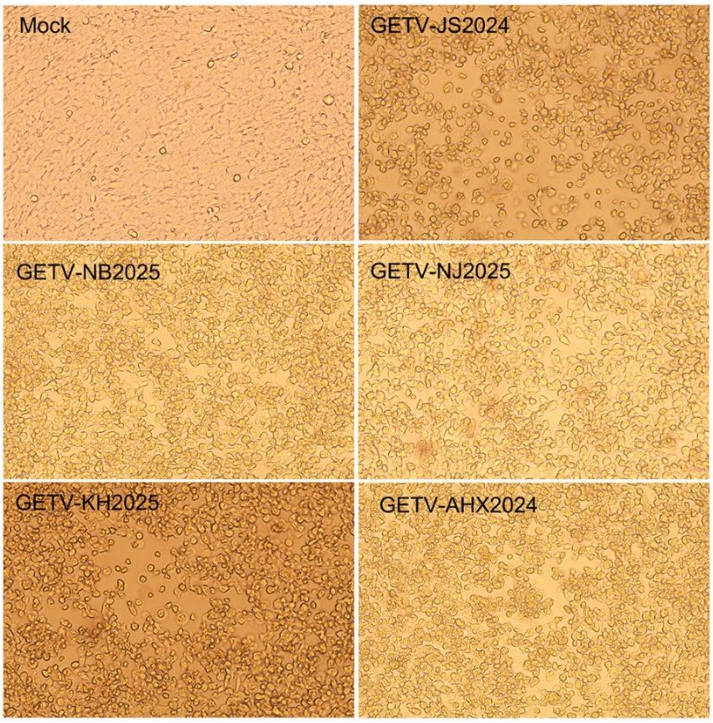

Figure S1 CPEs of GETV strains on BHK-21 cells.

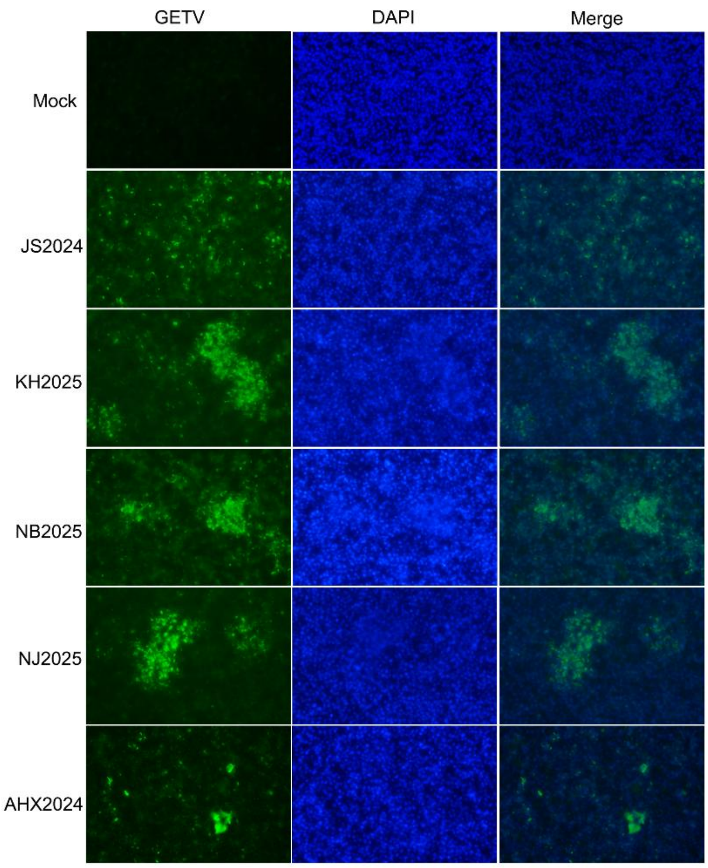

Figure S2 Indirect immunofluorescence of GETV strains on BHK-21 cells.

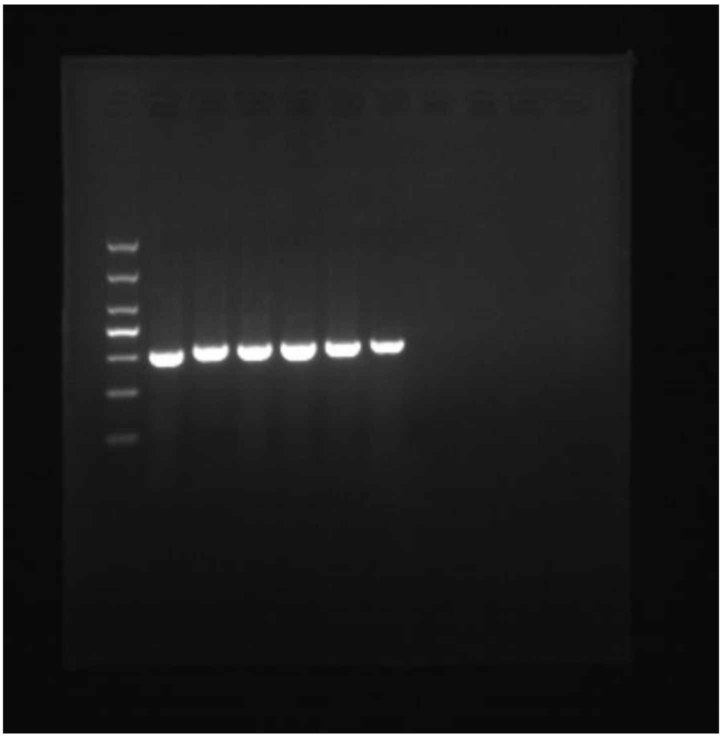

Figure S3 Original file for figure 2B.

Table S2 Genome characteristics of the GETV strains

Isolate	Genome length	GC content	ORF1	ORF2
AHX2024	11695	52.31%	7233	3684
JS2024	11694	52.32%	7233	3684
KH2025	11691	52.29%	7233	3684
NB2025	11689	53.33%	7233	3684
NJ2025	11691	52.28%	7233	3684

Table S3 GETV complete genome sequences collected from the GenBank database for phylogenetic analysis

GenBank no.	Strains	Collection date	location	Host
PV521858.1	GETV/mosq/ZJYW/2023	2023	China	mosquito
PV521859.1	GETV/mosq/ZJNX/2023	2023	China	mosquito
PV521860.1	GETV/mosq/ZJLH/2023	2023	China	mosquito
OP747412.1	GD2202	2022	China	mosquito
MW512827.1	NMDK1813-1	2018	China	mosquito
MH722255.1	JL1707	2017	China	mosquito
MG869691.1	JL17/08	2017	China	mosquito
LC107870.1	SC1210	2012	China	Armigeres subalbatus
KY434327.1	YN12031	2012	China	Armigeres subalbatus
EU01561.1	M1	2007	China	Mosquito
EU015063.1	YN0540	2008	China	Armigeres subalbatus
EU015062.1	HB0234	2007	China	Culex tritaeniorhynchus Giles
PX123982.1	JS202508	2025	China	Pig
PQ289630.1	GETV-QJ	2024	China	Pig
PQ658739.1	HNzmd-XP1	2024	China	Pig
PQ658750.1	HNsq-ZC	2024	China	Pig
PQ846024.1	GETV-JL	2024	China	Pig
PQ846025.1	GETV-HD	2024	China	Pig

PQ846027.1	GETV-HC	2024	China	Pig
PX312190.1	GETV-CX7	2024	China	Pig
PX207692.1	HNxy-24	2024	China	Pig
PV235474.1	GETV-WH	2024	China	Pig
PV455028.1	GETV-AHHB202410	2024	China	Pig
OR487192.1	GDHYLC23	2023	China	Pig
PQ034602.1	GETV-HeN202309	2023	China	Pig
PQ497971.1	CQ230629	2023	China	Pig
OQ968487.1	GETV-JX-CHN-22	2022	China	Pig
PP623164.1	SD2206	2022	China	Pig
PV615460.1	SD202205	2022	China	Pig
ON843770.1	GDJM2022	2022	China	Pig
ON987235.1	GDQY2022	2022	China	Pig
OL352731.1	GETV-YL	2021	China	Pig
OP004827.1	SC202010	2020	China	Pig
OP004828.1	SCZY202010	2020	China	Pig
MZ736796.1	GX1	2019	China	Pig
MK693225.1	SC201807	2018	China	Pig
MT269657.1	GX201808	2018	China	Pig
MN478486.1	SC483	2018	China	Pig
MN478487.1	SC266	2018	China	Pig
MG865968.1	HNPDS-1	2017	China	Pig
MG865965.1	AH9192	2017	China	Pig
MF741771.1	HuN1	2017	China	Pig
MG865966.1	HNNY-1	2016	China	Pig
KY399029.1	GETV-V1	2016	China	Pig
KY363862.1	HNJZ-S1	2011	China	Pig
MH722256.1	JL1808	2018	China	Cattle
OR371719.1	YN2305	2023	China	Cattle
MZ388464.1	GETV-XJ-2019-07	2019	China	Horse
PV089541.1	JX2024	2024	China	Boar
PP236766.1	JLy1	2023	China	Sus scrofa
PP537546.1	NM2022	2022	China	Sus scrofa
OM363683.1	BJ0304	2021	China	Sus scrofa
MZ736800.1	HeN2021	2021	China	Sus scrofa
OK423758.1	SC202009	2020	China	Sus scrofa
MZ736790.1	JX202004	2020	China	Sus scrofa
MZ736788.1	FJ202005-2	2020	China	Sus scrofa
MZ736795.1	GX201909	2019	China	Sus scrofa
MZ736797.1	GD201909	2019	China	Sus scrofa

MZ736793.1	HeB201707	2017	China	Sus scrofa
PQ816951.1	JXFZ2024	2024	China	Chicken
PQ816952.1	JXJA2024	2024	China	Chicken
OP593309.1	dog202206	2022	China	Dog
OR373097.1	GETV-China/GX2020	2020	China	pangolin
OP593308.1	Rbsq202206	2022	China	red-bellied tree squirrel
MH106780.1	SD17/09	2017	China	fox
EF631999.1	LEIV 17741 MPR	2007	Mongolia	Culex sp
LC814433.1	22IH8	2022	Japan	Mosquito
LC223130.1	16-I-599	2016	Japan	Equus caballus
LC223132.1	16-I-674	2016	Japan	Equus caballus
LC223132.1	16-I-676	2016	Japan	Equus caballus
LC212973.1	15-I-1105	2015	Japan	Sus scrofa
LC212972.1	15-I-752	2015	Japan	Sus scrofa
LC079088.1	14-I-605-C1	2014	Japan	Equus caballus
LC152056.1	12IH26	2012	Japan	Culex tri-taeniorhynchus
LC079087.1	MI-110-C2	1978	Japan	Equus caballus
MW410934.1	M 6-Mag 132		Japan	
AB032553.1	Sagiyama virus	1956	Japan	Mosquito
MN849355.1	MM2021	1955	Malaysia	Mosquito
LC534253.1	GETV/SW/Thailand/2017	2017	Thailand	Sus scrofa
AY702913.1	South Korea	2004	South Korea	Pig
EF631998.1	LEIV 16275 Mag	2007	Russia	Aedes sp.
